# Dominance of transposable element-related satDNAs results in great complexity of “satDNA library” and invokes the extension towards “repetitive DNA library”

**DOI:** 10.1007/s42995-024-00218-0

**Published:** 2024-04-11

**Authors:** Monika Tunjić-Cvitanić, Daniel García-Souto, Juan J. Pasantes, Eva Šatović-Vukšić

**Affiliations:** 1 Division of Molecular Biology, Ruđer Bošković Institute, 10000 Zagreb, Croatia; 2 Genomes and Disease, Centre for Research in Molecular Medicine and Chronic Diseases (CIMUS), Universidade de Santiago de Compostela, 15706 Santiago de Compostela, Spain; 3 Department of Zoology, Genetics and Physical Anthropology, Universidade de Santiago de Compostela, 15706 Santiago de Compostela, Spain; 4 Centro de Investigación Mariña, Dpto de Bioquímica, Xenética e Inmunoloxía, Universidade de Vigo, 36310 Vigo, Spain

**Keywords:** Satellitome, Comparative satellitomics, Helitron, Bivalves, “Dark matter of the genome”

## Abstract

Research on bivalves is fast-growing, including genome-wide analyses and genome sequencing. Several characteristics qualify oysters as a valuable model to explore repetitive DNA sequences and their genome organization. Here we characterize the satellitomes of five species in the family Ostreidae (*Crassostrea angulata, C. virginica, C. hongkongensis, C. ariakensis, Ostrea edulis*), revealing a substantial number of satellite DNAs (satDNAs) per genome (ranging between 33 and 61) and peculiarities in the composition of their satellitomes. Numerous satDNAs were either associated to or derived from transposable elements, displaying a scarcity of transposable element-unrelated satDNAs in these genomes. Due to the non-conventional satellitome constitution and dominance of Helitron-associated satDNAs, comparative satellitomics demanded more in-depth analyses than standardly employed. Comparative analyses (including *C. gigas*, the first bivalve species with a defined satellitome) revealed that 13 satDNAs occur in all six oyster genomes, with Cg170/HindIII satDNA being the most abundant in all of them. Evaluating the “satDNA library model” highlighted the necessity to adjust this term when studying tandem repeat evolution in organisms with such satellitomes. When repetitive sequences with potential variation in the organizational form and repeat-type affiliation are examined across related species, the introduction of the terms “TE library” and “repetitive DNA library” becomes essential.

**Supplementary Information:**

The online version contains supplementary material available at 10.1007/s42995-024-00218-0.

## Introduction

The ubiquitous but least understood DNA component of eukaryotic genomes is repetitive DNA sequences, also called the “dark matter of the genome” (Sedlazeck et al. 2018). These sequences are divided into two groups: (1) satellite DNAs (satDNAs) composed of arrays formed by sequences repeated in tandem; and (2) transposable elements (TEs) interspersed throughout the genome (Biscotti et al. 2015; Charlesworth et al. 1994; Jurka et al. 2007; López-Flores and Garrido-Ramos 2012; Schmidt and Heslop-Harrison 1998). Classical concept of satDNA organization presumes long arrays of hundreds to thousands of monomers that build heterochromatic blocks located at pericentromeric, subtelomeric, and interstitial chromosomal loci (reviewed in Garrido-Ramos 2017; Plohl et al. 2012; Thakur et al. 2021). SatDNAs and TEs are both builders of genome architecture and drivers of its evolution, as genome evolution is impacted by processes that reorganize repetitive DNA sequences and change their copy number (Biscotti et al. 2015; Garrido-Ramos 2017; Hartley and O’Neill 2019; Kojima 2019; Lopez-Flores and Garrido-Ramos 2012). SatDNAs and TEs are connected in various aspects. For example, multiple insertions of TEs into satDNA arrays occur, and such loci can serve as hotspots for further insertions (Palomeque et al. 2006; Šatović et al. 2016). Furthermore, satDNA repeats can arise through tandemization of TEs or its parts (Belyayev et al 2020; Biscotti et al. 2008; Langdon et al. 2000; Macas et al. 2009; McGurk and Barbash 2018; Sharma et al. 2013; Tek et al. 2005), and satDNA arrays can expand from short arrays found within the TEs (Dias et al. 2014; Luchetti 2015; Vondrak et al. 2020). The TEs of the Helitron/Helentron superfamily at their ends hold conserved sequence segments that incorporate subterminal inverted repeats, while in the central part they contain satDNA-like tandem repeats (Thomas and Pritham 2015). The examples of such elements in oysters include CvA, CvE, and CvG, which differ in nucleotide sequence but share structural characteristics (Gaffney et al. 2003). Some of the central repeats of Cg_HINE elements (Vojvoda Zeljko et al. 2020) are related to the most abundant satDNA of oysters Cg170/HindIII (Clabby et al. 1996; López-Flores et al. 2004).

Improvements in sequencing technologies and the accessibility of genomic datasets have provided insights into the repetitive fraction of the genomes (Athanasopoulou et al. 2022; Lower et al. 2018; Šatović et al. 2020; Sedlazeck et al. 2018). New software has enabled high-throughput analyses, large-scale detection and characterization of repeats, e.g., RepeatExplorer (Novák et al. 2010, 2013, 2020) and TAREAN (Novák et al. 2017). Not requiring genome assembly, these are especially valuable for studying repeats in non-model species.

The term “satellitome” describes all genomic satDNAs (Ruiz-Ruano et al. 2016), and the complete set of repetitive DNAs in the genome is called the “repeatome” (Pita et al. 2017). The above approaches are used to define satellitomes and repeatomes, and contribute to understanding evolutionary relationships among repetitive sequences in related species (reviewed in Šatović-Vukšić and Plohl 2023). One of the major postulates of satDNA evolution is the “library model” which posits that related species share a repertoire (library) of satDNAs inherited from a common ancestor. Any member of this library may undergo amplification and emerge as major satDNAs while others persist at lower levels, resulting in species-specific profiles (Fry and Salser 1977).

The “omics” studies on bivalve mollusks, accompanied by genome assemblies, are facilitating their use as models (Gomes-dos-Santos et al. 2020; Robledo et al. 2018; Suárez-Ulloa et al. 2013). Bivalves possess characteristics that make them useful models to explore repetitive DNA sequences, as exemplified by the data from the invasive Pacific oyster *C. gigas.* Among them are (1) scarce heterochromatin, limited to the centromeric region of a one chromosome pair and the telomeric region of another (Bouilly et al. 2008; Tunjić Cvitanić et al. 2020); (2) scarcity of satDNAs compared to TEs (Peñaloza et al. 2021; Zhang et al. 2012); (3) a high number of Helitron TEs (Peñaloza et al. 2021); (4) the incorporation of short satDNA arrays into TEs of the Helitron/Helentron family (Šatović et al. 2016; Vojvoda Zeljko et al. 2020); and (5) highly scattered organization of satDNA arrays across the genome (Šatović Vukšić and Plohl 2021; Tunjić-Cvitanić et al. 2021). As the latter contrasted with the classical concept (as outlined above), this represented a novel pattern of satDNA organization on the genome level (Tunjić-Cvitanić et al. 2021).

These characteristics have raised questions about satDNA composition and organization in oysters and the applicability of the satDNA library model. To this end, we characterize the satellitomes of five species from the Ostreidae family (*C. angulata, C. virginica, C. hongkongensis, C. ariakensis, Ostrea edulis*) and reveal their specificities. We then infer the relationships between the satellitomes (with the supplement of previously characterized *C. gigas* satellitome) and test the applicability of satDNA library model to this set of species. Following that, we investigate organizational forms of the arrays and divergence profiles for the most abundant satDNA in all species. In addition, we report the chromosomal distribution of several most prominent satDNA sequences of oysters.

Our results present novel and non-conventional satellitome constitution and we propose that the term “satDNA library” needs redefinition when studying repeat evolution in these organisms.

## Materials and methods

**Fig. 1:**
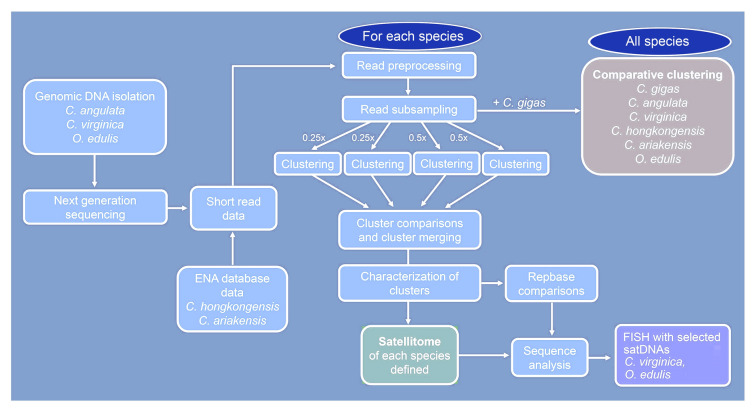
The workflow of activities employed in studying satellitomes in oyster species

Figure 1 outlines the strategies employed in this work.

### DNA isolation, barcoding and obtaining short-read sequencing data

Genomic DNA of *Crassostrea virginica*, *C. angulata*, and *O. edulis* were extracted from adductor muscle tissue using the DNeasy Blood and Tissue Kit (Qiagen). Molecular identification of species was performed using primers for the mitochondrial cytochrome c oxidase subunit 1 (LCO-1490 5′-GGT CAA CAA ATC ATA AAG ATA TTG G-3′ and HCO-2198 5′-TAA ACT TCA GGG TGA CCA AAA AAT CA-3′). PCR amplification was performed at 94 °C for 5 min, 35 cycles of: 94 °C for 30 s, 52 °C for 30 s, 72 °C for 30 s; followed by 72 °C for 10 min. PCR sequences were compared with those from the NCBI GenBank database. KAPA Hyper Prep library preparation using UDI-UMI adapters and next-generation sequencing (NGS) of genomic DNAs was performed by the Admera Health facility (USA) on an Illumina NovaSeq S4 platform. Pair-end sequencing generated 2 × 18,121,416 reads for *C. virginica*, 2 × 16,587,089 for *C. angulata* and 2 × 16,954,299 for *O. edulis*, read length 141 bp. The raw sequencing data were deposited in the Sequence Read Archive (SRA) database, under the BioProject accession numbers: SRR24520456 (*C. virginica*), SRR24523588 (*C. angulata*)*,* and SRR24523725 (*O. edulis*). Illumina short-read data for *C.* *hongkongensis* (accession number SRR12321640, read length 116 bp) and *C. ariakensis* (SRR14864893, read length 126 bp) were retrieved from the ENA database.

### SatDNA detection and comparative satellitomics

Genomic repeat identification was performed on the Galaxy server (https://repeatexplorer-elixir.cerit-sc.cz/galaxy/), employing the RepeatExplorer2 pipeline (Novák et al. 2013) with integrated TAREAN (Novák et al. 2017). Genomic reads of all species were quality filtered, trimmed, interlaced, and pair-end reads with no overlap used for further analyses. As low genome coverage (0.1–0.5 ×) is recommended for repetitive DNA analysis (Novák et al. 2017), subsets of reads were generated. To obtain satDNA totality, for each species four subsets were produced, two for the 0.25 × genome coverage and two to 0.5 × coverage. Similarity-based read clustering was performed. The results of the four analyses were combined to define satellitomes. Genome size for each species and the number of reads used for the analyses is presented in Table 1.

**Table 1:** Genome sizes of oyster species and the number of reads used for the analyses

Species	Genome size	Number of reads 0.25 × coverage	Number of reads 0.5 × coverage
*C. virginica*	684 Mb	1,221,424	2,442,858
*C. angulata*	590 Mb	1,053,572	2,107,142
*C. hongkongensis*	650 Mb	1,413,043	2,826,086
*C. ariakensis*	614 Mb	1,228,000	2,456,000
*O. edulis*	1.14 Gb	2,035,714	4,071,428

Nucleotide sequences constituting the satellitomes of five species defined in this work are available under the accession numbers: OQ989319–OQ989351 (*C. virginica*), OQ989352–OQ989413 (*C. angulata*), OQ989414–OQ989469 (*C. hongkongensis*), OQ989470–OQ989517 (*C. ariakensis*), OQ989518–OQ989570 (*O. edulis*). *Crassostrea gigas* satellitome is available under the accession numbers: OQ989571–OQ989623.

For comparative clustering, one dataset (corresponding to the 0.25 × genome coverage) was used for each species. For *C. gigas*, this corresponds to 1,053,572 reads. Consensus dataset of the satDNAs from the individual analysis of six species were used as a reference during comparative clustering, which enabled tracking of their distribution.

Additional comparative analysis was performed via RepeatProfiler (Negm et al. 2021). RepeatProfiler does not conduct simultaneous clustering of reads from all species, as RepeatExplorer2 does. RepeatProfiler generates read depth profiles by mapping reads from each species to the consensus sequences of RepeatExplorer2-obtained satDNAs. Consensus sequences were concatenated into dimers and used as references. Pair-end reads corresponding to 0.25 × genome coverage for each species were used. Analysis was run under the default parameters.

### Satellite DNA analysis

To identify clusters of the same satDNA across diverse datasets, we performed comparisons among satDNA sequences from four rounds of read clustering for each species. This was conducted by discontinuous megablast in Geneious Prime v.2023.1.1 software (Biomatters Ltd., Auckland, New Zealand). Local satDNA databases forming further inter and intra-species sequence comparisons and alignments were performed using the same software. SatDNA abundances were presented as an average from the four analyses.

Tandem organization of RepeatExplorer2-obtained satDNAs was checked on genome assemblies of the respective species, GenBank accessions: GCA_025612915.2 (*C. angulata*), GCA_002022765.4 (*C. virginica*), GCA_015776775.1 (*C. hongkongensis*), GCA_020567875.1 and GCA_020458035.1 (*C. ariakensis*), GCA_023158985.1 and GCA_947568905.1 (*O. edulis*). Consensus sequences of satDNA monomers were annotated on chromosomes and scaffolds in Geneious Prime, allowing 30% divergence from the consensus to encompass sequence variants.

We used RepeatMasker (https://www.repeatmasker.org/RepeatMasker/, version 4.1.3) to perform additional assessment of the prevalence of satDNAs identified by RepeatExplorer2. Randomly selected read pairs of 0.25 × coverage for each species were aligned to dimers of the respective satDNA consensus. The abundance was normalized by dividing the total length mapped to each satDNA by the genome length, following Cabral-de-Mello et al. (2023).

CENSOR was used to screen the query sequences against Repbase, a database of repetitive DNA sequences of eukaryotic species (Bao et al. 2015). Hits with less than 50% monomer coverage and < 70% identity were excluded.

### Cg170/HindIII analyses

For comparison of Cg170/HindIII between the six oyster species, RepeatExplorer2-obtained consensus sequences of this satDNA were dimerized and aligned. Subsequently, monomers of the same frame were extracted. A phylogenetic tree was constructed using the UPGMA method within the Geneious Prime software, using satDNA monomer consensus of each species.

To assess the sequence divergence, we extracted the reads belonging to Cg170/HindIII from the respective cluster for each species. The reads were mapped against the Cg170/HindIII consensus dimer of the respective species in Geneious Prime software. Divergence times between species were obtained by TimeTree (Kumar et al. 2022).

Analysis of the organizational forms of Cg170/HindIII satDNA across species was performed as described in Tunjić-Cvitanić et al. (2021). For this, the following genome assemblies were used: GCA_902806645.1 (*C. gigas*), GCA_025612915.2 (*C. angulata*), GCA_002022765.4 (*C. virginica*), GCA_015776775.1 (*C. hongkongensis*), GCA_020458035.1 (*C. ariakensis*), GCA_023158985.1 (*O. edulis*). A custom-made Python script was used to extract the sequence segments for each Cg170/HindIII array and its flanking regions (2000 base pairs on each side). The analysis used conserved Box1 and Box2 of Helitron elements, following Tunjić-Cvitanić et al. (2021). The annotation of boxes on the flanking regions was done in Geneious Prime. If the boxes were detected on each side of the array, arrays were classified as element-associated. Arrays having a box only on one side were considered as “intermediate” organizational form. If Helitron boxes were not detected, arrays were classified as standalone. Arrays of *C. gigas* Cg170/HindIII (CgiSat01) from Tunjić-Cvitanić et al. (2021), were included in this analysis. Here, arrays ranging from dimers to multimers were analyzed.

### Mitotic chromosomes preparations

Slides with mitotic metaphase chromosomes were prepared following protocols of Martínez-Expósito et al. (1994), with few modifications. *Crassostrea virginica* and *O. edulis* specimens were treated for 12 h in a 0.005% colchicine solution, followed by excision of gills. Bivalve gill tissue underwent hypotonic shock in seawater, followed by fixation in ethanol:acetic acid (3:1) for 1 h. Dissected gills were disaggregated with 60% acetic acid and the resulting cell suspensions dropped onto preheated slides (56 °C).

### Probe labelling

DNA probes for fluorescent in situ hybridization (FISH) were labelled by PCR. Each 50 µL reaction contained 50 ng of DNA, 2.5 U GoTaq Flexi G2 DNA polymerase (Promega), GoTaq Buffer, 1.5 mmol/L MgCl_2_, primers (1 µmol/L each), and either a dNTP mix with biotin-16-dUTP (Jena Bioscience) for satDNAs or digoxigenin-16-dUTP (NEB) for 28S rDNA controls. Probes were purified using the QIAquick PCR Purification Kit (Qiagen), validated on an agarose gel and quantified by Qubit Fluorometer. Nucleotide sequences of the primers and PCR amplification conditions are provided in Supplementary Table S6.

### Fluorescent in situ hybridization

FISH experiments were performed following the protocol of Pérez-García et al. (2011), with the modification in pepsin digestion to 5 min at 37 °C. For FISH experiments metaphase chromosomes of triploid *C. virginica* (*3n* = 30) and diploid *O. edulis* (*2n* = 20) were used. The 28S rDNA was used as a positive control to confirm signal specificity. Probes were denatured for 8 min at 80 °C and placed on ice for 2 min. The 50 ng of each probe was used. Signal detection was carried out with fluorescein-labelled avidin (Vector) diluted 1:200, biotinylated anti-avidin (Vector) 1:100, and fluorescein-labelled avidin 1:200 for the biotinylated probes. For the digoxigenin-labeled probes mouse anti-digoxigenin (Sigma-Aldrich) 1:500, goat anti-mouse rhodamine (Sigma-Aldrich) 1:200, and rabbit anti-goat rhodamine (Sigma-Aldrich) 1:100 were used, following instructions of the supplier. The counterstaining of the chromosomes was performed using 100 ng/mL DAPI (Sigma-Aldrich), and slides were subsequently mounted with VECTASHIELD (Vector) antifade medium. Fluorescent microscopy was used for signal visualization and image capturing.

## Results

### Satellitome analysis

Results of RepeatExplorer2 clustering on four subsampled sets for each species are presented in Supplementary file 1. The satellitomes derived from them are presented in Supplementary Tables S1–S5.

In the genome of *C. angulata*, 61 satDNAs were detected, which make 7.70% of the genome (Supplementary Table S1). The detected satDNAs exhibited a broad range of monomer lengths, from 28 bp (CanSat61) to 8637 bp (CanSat15). A total of 51 satDNAs, which make up 7.32% of the genome and 95.17% of the satellitome, showed similarity to some of the repetitive DNAs from RepBase. Noteworthy, 15 of them, constituting 4.95% of the genome and 64.28% of the satellitome, presented similarity to the central repeats of Helitron TEs (Supplementary Table S1). The most abundant satDNA in the genome was CanSat01. Blast search disclosed its correspondence to the Cg170/HindIII (reported by Clabby et al. 1996; López-Flores et al. 2004) and CgiSat01 satDNA of *C. gigas* (reported by Tunjić-Cvitanić et al. 2021). This satDNA constituted 1.32% of *C. angulata* genome, which is 17.11% of its satellitome. In this species, as well as in the genome of the Pacific oyster *C. gigas*, it was found in two variants, CanSat01a and b. The two variants display nucleotide divergence in short stretches within the monomer sequence (pairwise identity 82%) and differ in the monomer length, 164 bp (CanSat01a) and 166 bp (CanSat01b).

**Fig. 2:**
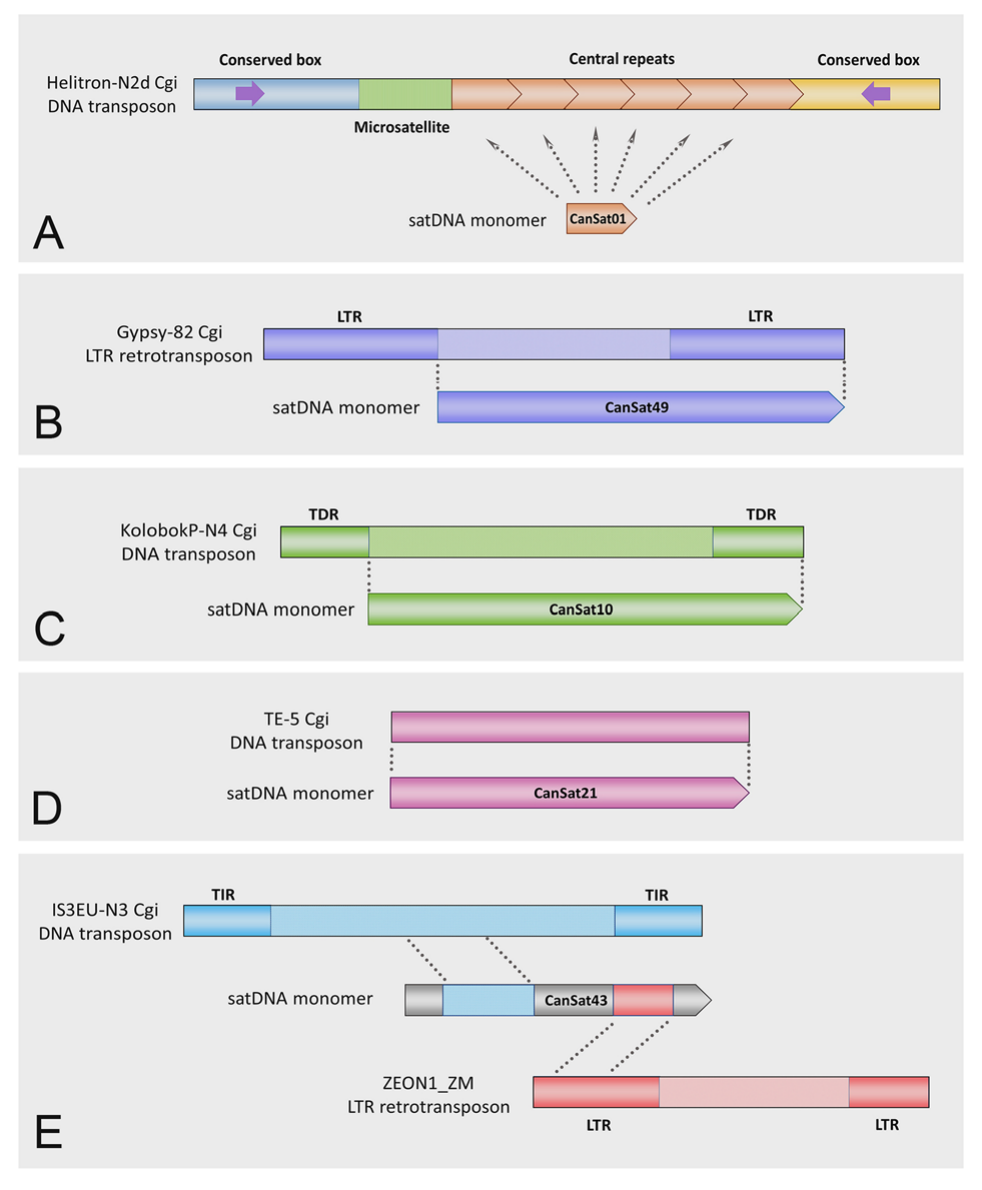
The sequence similarities between satDNAs and transposable elements or their constitutive parts, exemplified by *C. angulata* satDNAs: **A** CanSat01, **B** CanSat49, **C** CanSat10, **D** CanSat21, **E** CanSat43. *LTR*  long terminal repeats, *TDR *terminal direct repeats, *TIR *terminal invert repeats. Subterminal inverted repeats found within conserved boxes of Helitron elements are represented by purple arrows

In all species inspected in this work, when satDNA monomer sequences exhibited similarity to Helitrons, this corresponded to the central repeats of these elements (Fig. 2A). For other types of TEs, monomer sequence corresponded to the entire element, part of the TE, or segments of the monomer sequence corresponded to the parts of different TEs (e.g., Fig. 2). When segments of the monomer sequence corresponded to the parts of different TEs (Fig. 2E), the Repbase search result was marked as “Multiple TEs” (Supplementary Tables S1–S5). The outputs of the RepBase searches can be found in Supplementary file 4.

**Fig. 3:**
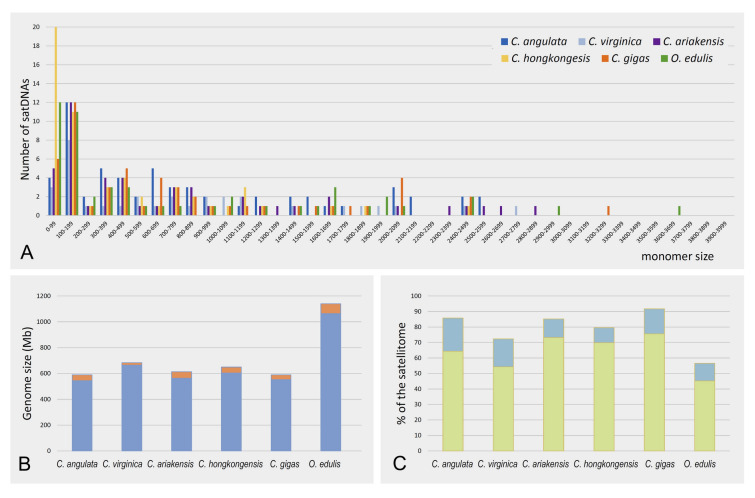
General features of satellitomes of six oyster species. **A** The abundance of satDNAs repeat in respect to monomer sizes (for better distinguishability, three satDNAs with the monomer size exceeding 4000 bp were omitted from the image (CanSat15, CanSat26 and OedSat26)). **B** The proportion of satDNAs constituting the satellitome (orange) in respect to the genome size of each oyster species. **C** The proportion of satellitome showing similarity to Helitron TEs (green) and the proportion of satellitome showing similarity to other TEs (blue)

A total of 33 satDNAs were detected in the genome of *C. virginica*, occupying 2.92% of the genome (Supplementary Table S2). The most represented satDNA was CviSat01, with a monomer length of 436 bp and genome share of 0.58%. Monomer lengths within the satellitome of this species ranged from 39 bp (CviSat29) to 2748 bp (CviSat31). However, among the satDNAs identified through RepeatExplorer2, the presence of Cg170/HindIII, corresponding to the most abundant satDNAs in *C. angulata* (CanSat01) and *C. gigas* (CgiSat01) was not initially detected in the genome of *C. virginica*. Upon further examination of clusters, encompassing both classified and unclassified ones, a variant of Cg170/HindIII was discovered within the predominant unclassified one. It constituted 1.5% of the *C. virginica* genome. The similarity search disclosed that this cluster corresponded to a TE of *C. virginica*, CvA, described by Gaffney et al. (2003). The *C. virginica* variant of Cg170/HindIII aligned with the central repeats of this element. Likewise, CviSat03 satDNA corresponded to the central repeats of CvE element and CviSat05 corresponded to the central repeats of CvG. Fifteen satDNAs of *C. virginica* exhibited similarity to elements from in Repbase, representing 2.12% of the genome and 72.3% of the satellitome. Six of satDNAs demonstrated similarity to Helitron TEs, constituting 1.6% of the genome and 54.31% of the satellitome (Fig. 3C).

Upon four rounds of RepeatExplorer2 clustering, 56 satDNAs were detected in the genome of *C. hongkongesis* (Supplementary Table S3). They constitute 7.14% of the genome of this species, and their monomer sizes range from 34 bp (ChoSat46) to 2421 bp (ChoSat23). In this species, the most abundant satDNA ChoSat01 is homologous to Cg170/HindIII. ChoSat01 occupied 0.98% of the genome and 13.65% of the satellitome. A total of 22 satDNAs showed similarity to the sequences from Repbase (5.7% of the genome, 79.54% of the satellitome), with 14 being associated to Helitron TEs (69.86% of the satellitome).

The satellitome of *C. ariakensis* constitute 51 satDNAs, which build 8.21% of the genome (Supplementary Table S4). The most prevalent, CarSat01, constituted 1.15% of the genome and 14% of the satellitome. It corresponded to the Cg170/HindIII satDNA. Monomer size range for this species varied between 42 bp (CarSat51) and 2823 bp (CarSat40). Thirty satDNAs showed similarity to TE within RepBase, among which Helitron TE dominate, comprising 73.23% of the satellitome.

In the genome of *O. edulis*, 52 satDNAs were detected by RepeatExplorer2 (Supplementary Table S5), comprising 6.70% of its genome. The most abundant satDNA in the genome is OedSat01, belonging to the Cg170/HindIII family of repetitive DNAs. Its genome share is 1.3%, the highest among the inspected set of species. Monomer size range for this species varied between 23 bp (OedSat43) and 16,346 bp (OedSat26). The unusually large size of OedSat26 indicates that it is a large tandemly repeated fragment of the genome rather than a conventional satDNA. Eighteen of the detected satDNAs (3.79% of the genome and 56.53% of the satellitome) presented similarity to TEs from Repbase. Eleven of them, with a total share of 3.03% in the genome, and 45.19% in the satellitome, presented similarity to Helitron TEs (Supplementary Table S5).

Properties of the satellitomes of the five inspected oyster species, with the addition of the data for *C. gigas*, are presented in Table 2 and Fig. 3. Repeat lengths of satDNAs varied extensively, but the majority was within the common range of repeat lengths, below 200 bp (Fig. 3A). *Crassostrea hongkongensis* had high number of satDNAs with monomer sizes below 100 bp. Despite variations in genome size (Fig. 3B), satellitomes made up ~ 6–8% of the genome across the species, except for *C. virginica* which exhibits a reduced genome occupancy at ~ 3% (Table 2). Substantial parts of the satellitomes exhibited similarity to different types of TE (Fig. 3C). In all the species Helitron-related satDNAs predominated. Only in the satellitome of *O. edulis* contribution of TE-related satDNAs was diminished, compared to species of *Crassostrea* (Fig. 3C).

**Table 2:** Summary of the main properties of oyster satellitomes

Species	Number of satellite DNAs	Monomer length range (bp)	Genome proportion (%)		Satellitome proportion (%)	Number of satDNA showing similarity to TE	Repbase similarities for satDNAs				
			Range	Total	Range		DNA/Helitron	other DNA transposons	Retrotransposons	Multiple TEs	Other
*C. angulata*	62	28–8637	0.01–0.72	7.70	0.13–9.32	39	15	13	4	5	4
*C. virginica*	33	39–2748	0.01–0.58	2.92	0.30–19.78	15	5	6	0	4	1
*C.hongkongensis*	56	34–2421	0.01–0.98	7.14	0.14–13.65	22	14	7	0	1	1
*C. ariakensis*	51	42–2823	0.01–1.15	8.21	0.12–14.01	29	14	12	2	1	3
*O. edulis*	52	23–16,346	0.01–1.30	6.70	0.18–19.48	18	11	4	1	2	0
C. gigas*	52	21–3287	0.01–0.72	6.33	0.16–11.29	38	16	16	6	0	2

*Data from Tunjić-Cvitanić et al. (2021), added for comparison

### Comparative satellitomics

A summary of the comparative clustering is presented in Supplementary Fig. S1, from which satDNAs distribution was followed. Comparative clustering resulted in a complex network of clusters (Supplementary file 3). We identified connections between sequences using the document with satDNAs from individual satellitomes’ analyses (Supplementary file 3). For instance, CviSat08, CanSat02, CgiSat02, OedSat05, CarSat02, and ChoSat03 were identified as members of the same satDNA family. The reads belonging to this satDNA were distributed throughout several clusters (Cl 12, 15, 16, 22, 60, 191, 377), with different representation of reads belonging to each species in each cluster (Supplementary file 3). Two of those (Cl 60 and 191) contained reads only from *C. virginica* and *O. edulis*. Clusters 22 and 377 comprised from reads belonging to CviSat08, added further complexity. These clusters either contained no (Cl 377) or very few reads (Cl 22) from other species and represented species-specific variants.

Cg170/HindIII is represented by CanSat01, CgiSat01, OedSat01, CarSat01, ChoSat01, and central repeats of CvA. Reads belonging to this satDNA from *C. angulata*, *C. gigas*, *O. edulis*, *C. ariakensis* and *C. hongkongensis* are present in Cl 1 (classified as satDNA) and Cl 19 (unclassified) (Supplementary file 3). *Ostrea edulis* reads that differ from the others clustered separately (Cl 6). Reads belonging to this satDNA were also present in the unclassified Cl 2, which unifies reads from all six species.

In Cl 64, only reads from *C. ariakensis* were recognized as CarSat20, while the reads of *C. angulata*, *C. gigas*, and *C. hongkongensis* within the same cluster indicated its presence in other species. Examples of clusters where a specific satDNA was classified in some but remained unclassified in others during individual satellitome analyses can be found throughout Supplementary file 3.

From the complex network of clusters, it was inferred that 13 satDNA were present in all six oysters, named OYS 1–13 (Table 3). OYS1 family represented Cg170/HindIII satDNA and was dispersed through series of classified and unclassified clusters. Some clusters contained reads from all species, while others only from subset of species, as elaborated. OYS2 was constituted by CviSat08, CanSat02, CgiSat02, OedSat05, CarSat02, and ChoSat03. It was distributed along seven clusters, in a previously explained manner. OYS3–5 each confined to a single cluster, with all reads attributed to a certain satDNA in course of individual satellitome definition. OYS6 was found in two clusters. First one was classified as satDNA (Cl 30), holding repeats from all six species (CviSat09, CanSat20, CgiSat14, OedSat51, CarSat22, ChoSat12). The second one was unclassified (Cl 166) and incorporated only those from *O. edulis*. OYS7 was distributed into two unclassified clusters, one holding repeats from all six species and the other omitting those from *C. virginica* and *O. edulis*. OYS8–13 were found each within one cluster and contained reads from all species.

**Table 3:** SatDNAs present in all six oyster species, adapted from Supplementary file 3

satDNA	Cl	RE classification	Total reads	Cvi reads	Can reads	Cgi reads	Oed reads	Car reads	Cho reads	Cvi	Can	Cgi	Oed	Car	Cho
OYS1	1	satDNA	60,379	0	16,851	15,571	64	14,171	13,722	CvA	CanSat01	CgiSat01	OedSat01	CarSat01	ChoSat01
	2	Unclassified	43,759	19,067	3243	2677	8667	4642	5463						
	6	satDNA	27,736	6	5	2	27,682	11	30						
	19	Unclassified	16,430	0	3530	2942	5	5134	4819						
OYS2	12	satDNA	19,886	0	4097	4546	0	5659	5584	CviSat08	CanSat02	CgiSat02	OedSat05	CarSat02	ChoSat03
	15	satDNA	18,740	2	4457	3679	1	3809	6792						
	16	satDNA	17,209	1	2342	2305	0	7373	5188						
	22	satDNA	14,946	14,941	2	0	3	0	0						
	60	satDNA	8477	1555	0	0	6922	0	0						
	191	satDNA	3258	2185	0	0	1073	0	0						
	377	satDNA	1567	1567	0	0	0	0	0						
OYS3	4	Unclassified	40,667	893	6425	6292	593	13,276	13,188	CviSat10	CanSat08	CgiSat05	OedSat05	CarSat04	ChoSat05
OYS4	85	satDNA	6376	101	547	779	3860	471	618	CviSat22	CanSat30	CgiSat16	OedSat10	CarSat36	ChoSat23
OYS5	148	satDNA	4226	512	613	578	1213	702	608	CviSat03	CanSat29	CgiSat52	OedSat29	CarSat29	ChoSat34
OYS6	30	satDNA	12,667	5915	1035	1138	56	1262	3261	CviSat09	CanSat20	CgiSat14	OedSat51	CarSat22	ChoSat12
	166	Unclassified	3882	0	0	0	3882	0	0						
OSY7	88	Unclassified	6288	1071	1280	863	1104	1233	737					CarSat12	ChoSat19
	91	Unclassified	6059	0	2847	1403	0	1131	678						
OYS8	55	Unclassified	8762	202	1746	1505	803	2102	2404	CviSat29	CanSat18	CgiSat38	OedSat40		
OYS9	62	Unclassified	8253	2237	1693	1163	575	1073	1512		CanSat15				
OYS10	87	Unclassified	6297	4358	237	181	902	284	335	CviSat32					
OYS11	389	satDNA	1488	525	173	236	276	48	230	CviSat20	CanSat51	CgiSat26			ChoSat48
OYS12	453	Unclassified	1124	391	187	278	44	78	146		CanSat59				
OYS13	515	satDNA	888	96	175	176	65	165	211		CanSat53	CgiSat27		CarSat46	ChoSat43

Listed are: cluster number in comparative clustering, RepeatExplorer2 classification of the cluster, total number of reads in the cluster, the number of reads contributed by each species, correspondence to a specific satDNA detected during the individual clustering

Additional comparative analysis was conducted via RepeatProfiler pipeline and read depth profiles are presented in Supplementary file 5. Reads corresponding to some satDNAs (e.g., CanSat16, CanSat19, CanSat43, CgiSat09, ChoSat04, ChoSat40) covered the consensus sequence in full length in several species, whereas in the remaining they mapped only to some segments (Supplementary file 5). This method did not identify any additional satDNA shared among all species.

### Cg170/HindIII satDNA

**Fig. 4:**
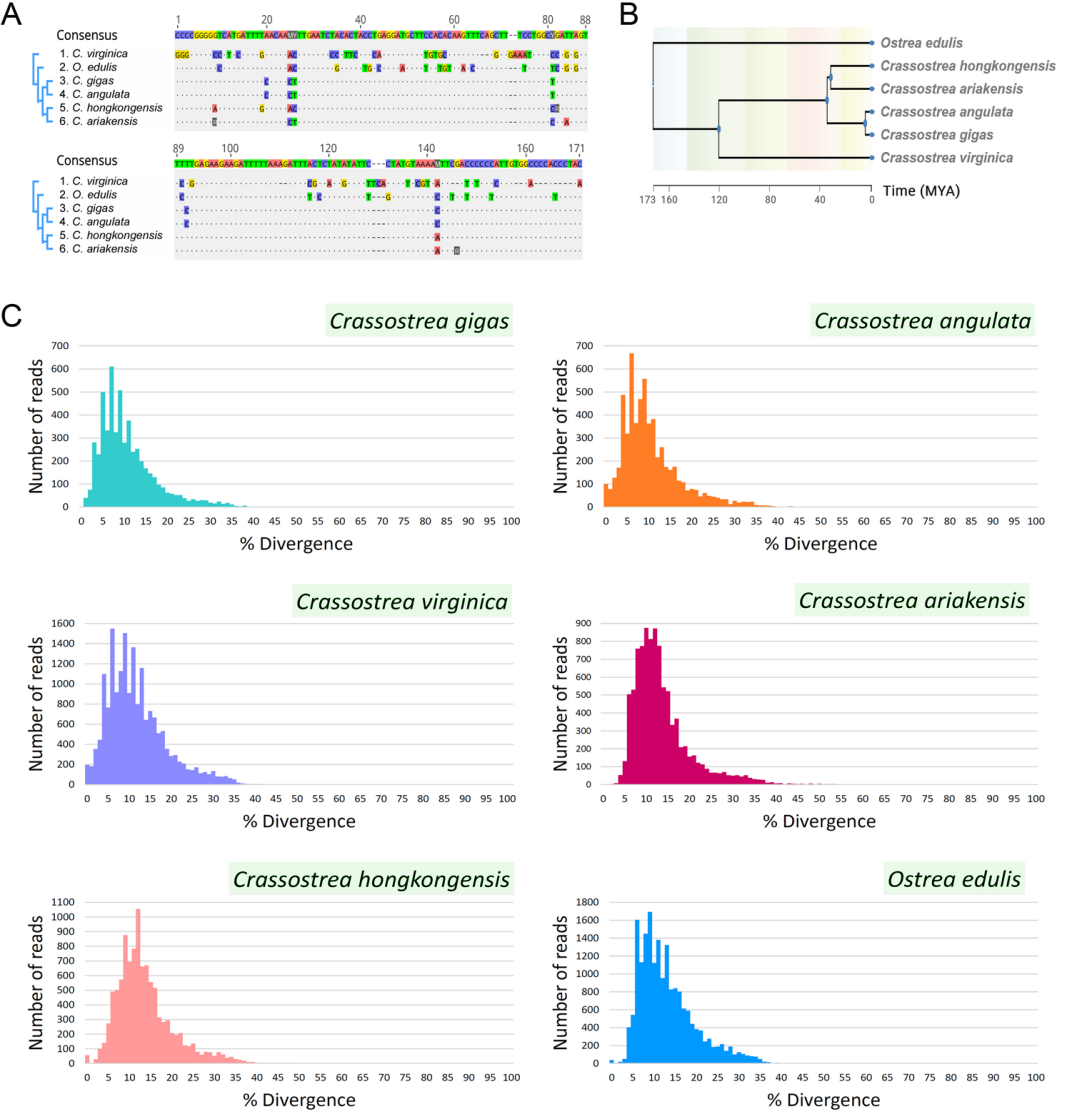
The properties of Cg170/HindIII satDNA in oyster species. **A** Alignment of the consensus sequences of Cg170/HindIII satDNA from six oyster species. **B** Time tree presenting separation times of inspected oyster species. **C** Divergence plots of Cg170/HindIII sequence in six oyster species

The most abundant tandem repeat of all species is Cg170/HindIII satDNA (OYS1), raising the interest for understanding the evolutionary processes shaping this sequence in oyster genomes. Sequence similarity of the Cg170/HindIII mirrored the evolutionary distance for *C. angulata, C. gigas, C. ariakensis, C. hongkongensis*, and *O. edulis*. However, Cg170/HindIII of *C. virginica*, has diverged significantly (Fig. 4A, B).

Following this, we generated divergence profiles of this satDNA across species. The landscapes of six species presented similar distribution (Fig. 4C). This was accompanied by comparable average divergence values: *C. gigas* 11%, *C. angulata* 11%, *C. virginica* 12%, *C. ariekensis* 13%, *C. hongkongensis* 14%, *O. edulis* 13%.

**Fig. 5:**
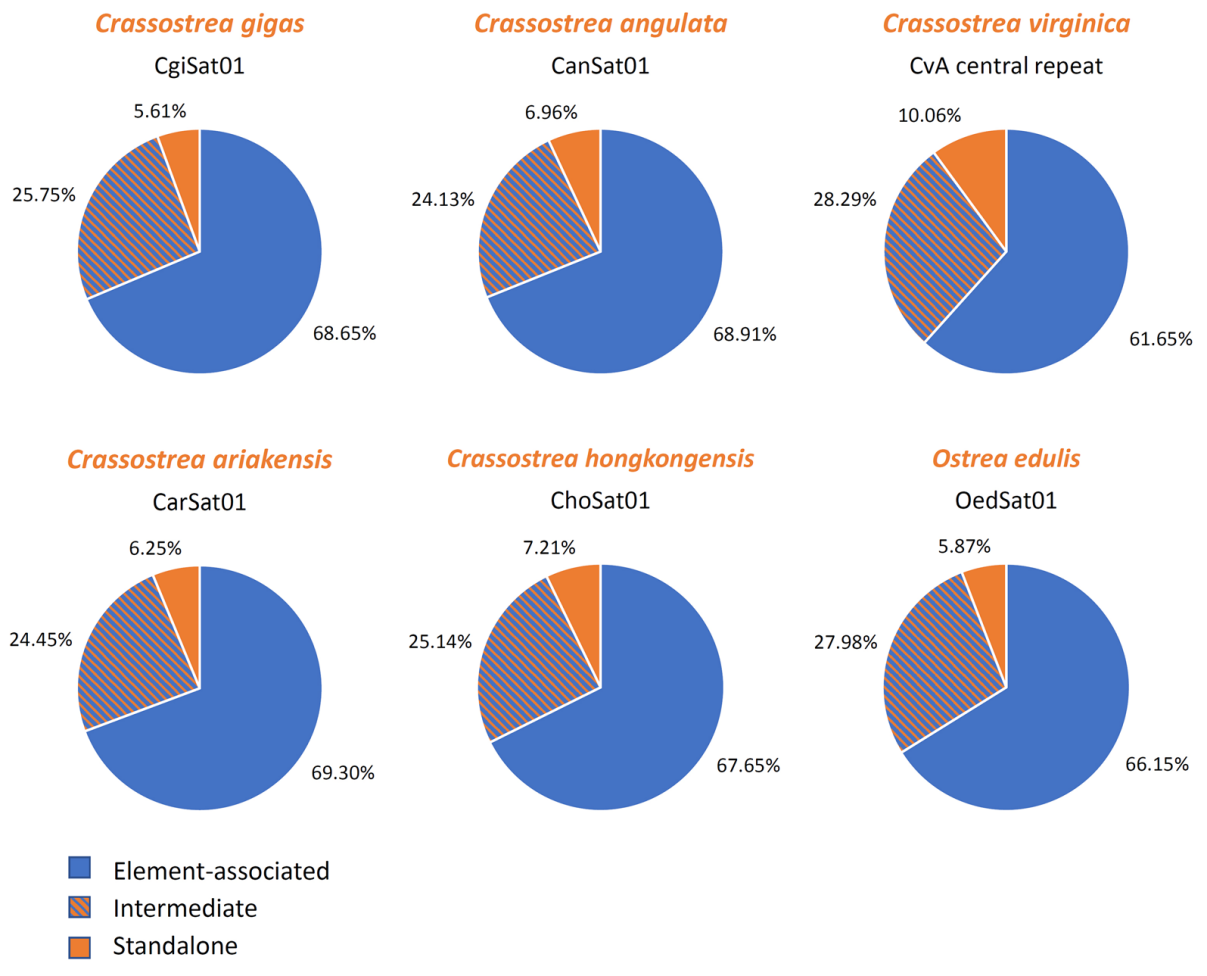
The proportion of element-associated, intermediate, and standalone organizational forms of Cg170/HindIII arrays across species

In continuation, we explored the genomic organization of Cg170/HindIII. We sought to ascertain the proportion of element-associated, intermediate, and standalone organizational forms. The number of analyzed arrays, and their affiliation with a specific organizational form is presented in Table 4. All three forms exist in all species, with TE-association being the dominant form of this sequence in all six oysters (Fig. 5). The intermediate form constituted 24–28% of the arrays. The classical standalone organization of satDNAs was displayed by 5–10% of the arrays.

**Table 4:** Organizational forms of Cg170/HindIII arrays across six oyster species

	*C. gigas*	*C. angulata*	*C. virginica*	*C. ariakensis*	*C. hongkongensis*	*O. edulis*
TE-incorporated	3661	3790	8451	5711	3668	8704
Intermediate	1373	1327	3878	2015	1363	3681
Standalone	299	383	1379	515	391	773
Number of arrays analyzed	5333	5500	13,708	8241	5422	13,158

### FISH

**Fig. 6:**
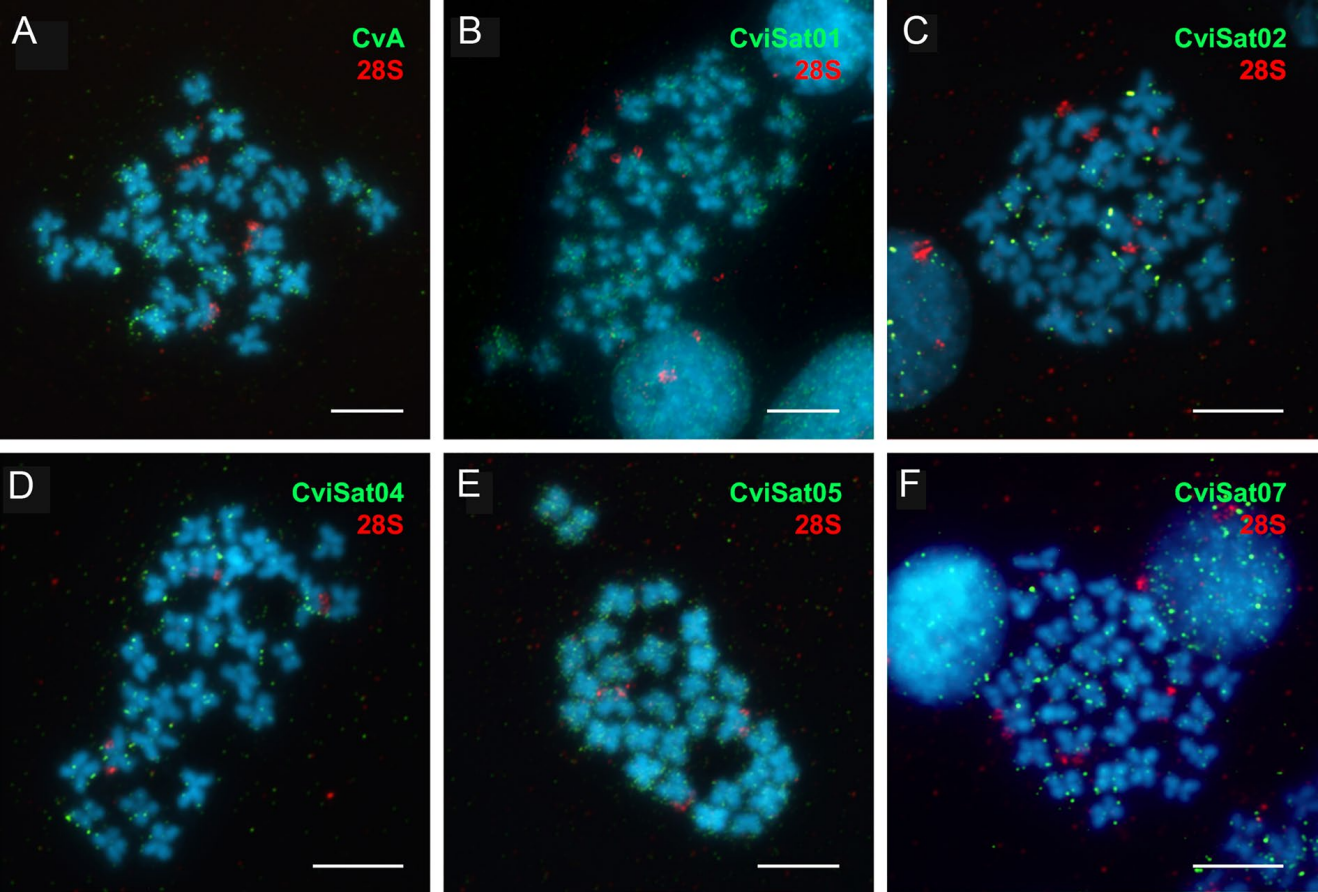
FISH mapping of *C. virginica* tandem repeats (green) of the following elements: **A** central repeats of CvA element, **B** CviSat01, **C** CviSat02, **D** CviSat04, **E** CviSat05, **F** CviSat07, and 28S rDNA positive control (red). Scale bar represents 5 µm

We performed FISH analysis on *C. virginica* and *O. edulis*, accessible for cytogenetic analysis. In *C. virginica*, probes for central repeats of CvA element, CviSat01, and CviSat05, displayed a substantial number of signals along the chromosome arms in a highly interspersed pattern (Fig. 6A, B, E). CviSat04 and CviSat07 satDNAs also exhibited interspersed pattern, but with a reduced number of signals (Fig. 6D, F). CviSat02 exhibited a combination of scattered weak signals and more pronounced clustered signals (Fig. 6C).

**Fig. 7:**
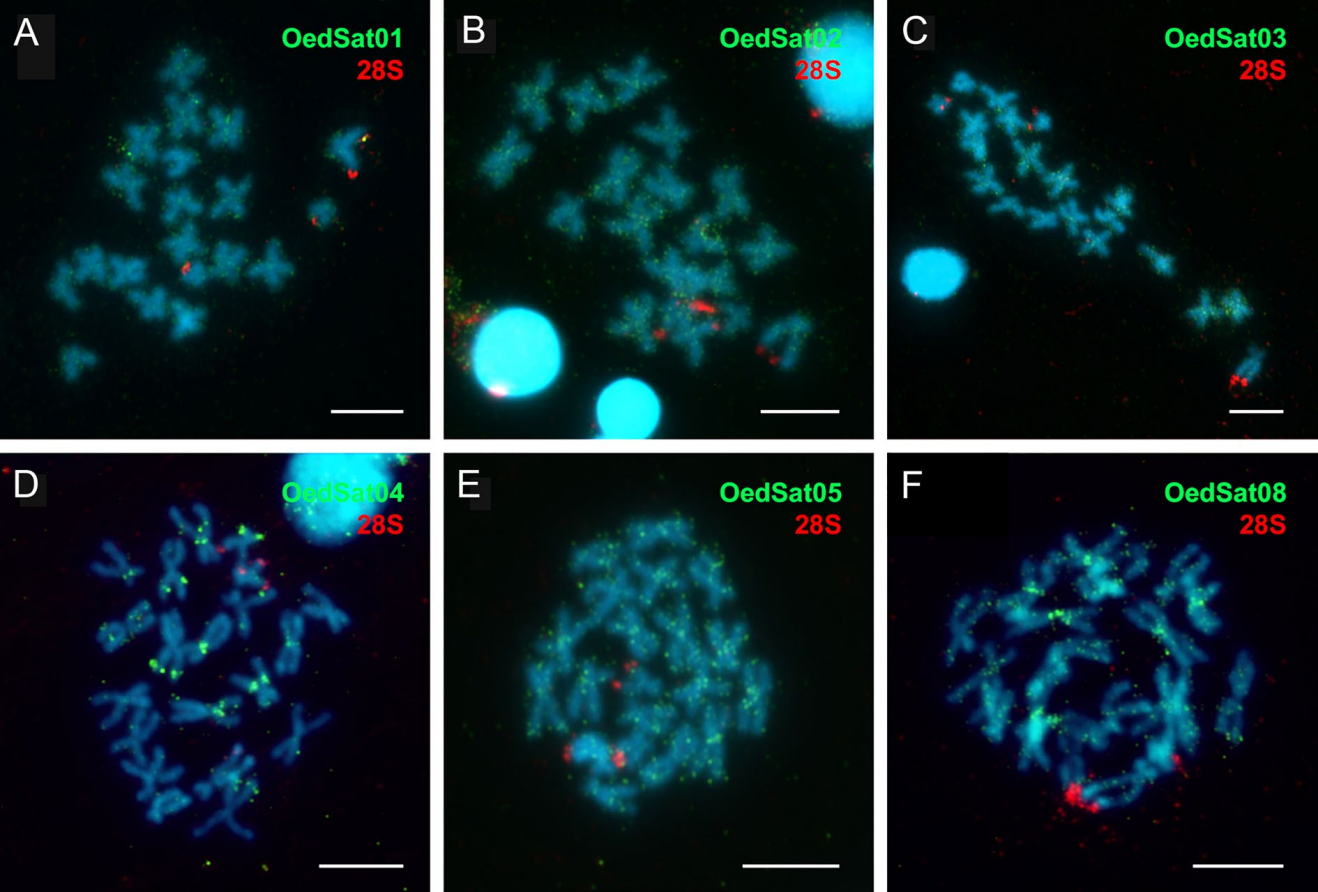
FISH mapping of *O. edulis* satDNAs (green): **A** OedSat01, **B** OedSat02, **C** OedSat03, **D** OedSat04, **E** OedSat05, **F** OedSat08, and 28S rDNA positive control (red). Scale bar represents 5 µm

SatDNAs OedSat01 and OedSat02 of *O. edulis* present highly interspersed signal distribution along the chromosomes (Fig. 7A, B). OedSat03 satDNA signals accumulate in the pericentromeric area of the majority of chromosomes (Fig. 7C). A reduced number of weak interspersed signals in combination with distinct (peri)centromeric clustering of signals was displayed by OedSat08 (Fig. 7F). For OedSat04, interspersed signals were accompanied with pericentromeric and subtelomeric signal clustering (Fig. 7D). OedSat05 presented a substantial number of signals along the chromosome arms, along with pericentromeric clustering of the signal (Fig. 7E).

## Discussion

A widely adopted strategy for detecting the inventory of repetitive DNAs without the need for a genome assembly was developed by Novák et al. (2013, 2017). It enabled the characterization of satellitomes and repeatomes in a number of species (reviewed in Šatović-Vukšić and Plohl 2023) and was a method of choice in this work. The overall number of satDNAs in oyster genomes is substantial, ranging from 33 in *C. virginica* to 61 in *C. angulata* (Supplementary Tables S1–S5). However, their overall genome contribution is low, amounting 6–7% in all species, except in *C. virginica*, being half as much. The number of satDNAs in the eukaryotic genomes and their genome share varies substantially among species (Šatović-Vukšić and Plohl 2023). The highest number of satDNAs identified is 258 families in the crayfish *Pontastacus leptodactylus* (Boštjančić et al. 2021), and the lowest is in the moth *Cydalima perspectalis* (one family, Cabral-de-Mello et al. 2021). The lowest genome contribution of a satDNA was 0.06% (the moth *Diatraea postlineella,* Cabral-de-Mello et al. 2021), and the highest 50.43% is in the olive *Olea europaea cuspidata* (Mascagni et al. 2022).

Experimental support of the “satDNA library model” proposed by Fry and Salser (1977) was achieved when examining numerous species using traditional research methods. These methods involve restriction enzyme-based detection of a satDNA, followed by the inspection of its presence in congeneric species (reviewed in Plohl et al. 2012). Investigating the “satDNA library” using the entire satellitomes is challenging, with outcomes largely depending on the experimental system (reviewed in Šatović-Vukšić and Plohl 2023). Here, the analysis of satellitomes and of satDNA library of oyster species from *Crassostrea* and *Ostrea* genera proved to be more complex than standard. This is due to the substantial number of satDNAs within each satellitome and the abundance of TE-related satDNAs. The substantial portion of each inspected satellitome was found to be connected with various TEs, particularly Helitrons (Supplementary Tables S1–S5, Tunjić-Cvitanić et al. 2021). Helitrons harboring tandem repeats in their central part contribute extensively to repeat misclassification/non-classification (Šatović-Vukšić and Plohl 2021; Tunjić Cvitanić et al. 2020). For example, during the RepeatExplorer2 analysis of *C. gigas*, tandem repeats originating from the central regions of Helitrons were grouped into a single cluster and classified as satDNA. Sequences corresponding to the conserved segments from ends of the element were assigned to two distinct clusters that remained unclassified. In other cases, the complete element was placed in an unclassified cluster (Šatović-Vukšić and Plohl 2021). Therefore, sequence recognized and characterized in the genome of one oyster as satDNA, and lacking from the RepeatExplorer2-produced satellitome of the other, may exist in the genome of the related species. An example is Cg170/HindIII in *C. virginica,* situated within the unclassified cluster. This emphasizes the necessity to employ individual clustering prior to employing a comparative one, the usage of referent document containing satellitome data of all species, and intensive manual curation to study satellitomes in these organisms. From the output of the comparative clustering (Supplementary Fig. 1) it might be concluded that a number of species-exclusive repetitive sequences existed in *C. virginica* and *O. edulis*. However, cluster analysis with the use of a data from the individually obtained satellitomes, identified some of them as species-specific variants of the shared sequence (Supplementary file 3).

Our analysis revealed that 13 satDNAs are shared by all six oysters OYS1-13 (Table 3). Their presence in all oyster species suggests the origin of 13 satDNAs from a common ancestor of both *Crassostrea* and *Ostrea*. This indicates that the minimum age of satDNAs is 173 million years ago (MYA), corresponding to the divergence time of these genera (Fig. 4B; Li et al. 2021). While the library model allows differential amplification of any satDNA in each of the related species (Fry and Salser 1977), OYS1/Cg170/HindIII profiled as the most abundant in all six species. In the genomes of *C. angulata* (Supplementary Table S1) and *C. gigas* (Tunjić-Cvitanić et al. 2021) this satDNA presented two variants (166 and 164 bp monomers), while others harbored only the 166 bp variant. Thus, the 164 bp subvariant emerged after the separation of the *Crassostrea* branch but before the divergence of *C. angulata* and *C. gigas*, which occurred between 30 and 3 MYA (Fig. 4B). In *C. virginica,* the sequence with the closest resemblance to Cg170/HindIII is a central repeat of the CvA element (Gaffney et al. 2003). However, this sequence differed in size and contained numerous mutations in respect to the consensus sequences from other species.

Divergence profiles provide insights into satDNA sequence variants. Peaks at lower divergence values result from recent amplification and/or homogenization process, while those at higher divergence values are older variants degenerated by the accumulation of mutations. Despite the differences observed in *C. virginica* Cg170/HindIII, its divergence profile corresponds to those of other species (Fig. 4C). Similarities in divergence profiles are not presumed, as evolution of a satDNA is influenced by various molecular mechanisms in each species (Camacho et al. 2022; Dover 1986; Garrido-Ramos 2017; Plohl et al. 2008, 2012; Thakur et al. 2021). The observed pattern of distribution (Fig. 4C) may reflect the shared organizational forms of these sequences, as their connection with Helitron TEs is evident in all species (Supplementary Tables S1–S5; Table 4; Fig. 5).

In the genome of the Pacific oyster *C. gigas* we have revealed an unusual, highly scattered organization of relatively short satDNA arrays throughout the genome (Tunjić-Cvitanić et al. 2021). Similarly, chromosomal mapping of satDNA in the insect *Oxycarenus hialinipennis* revealed its high spread in the euchromatic regions (Cabral-de-Mello et al. 2023). These findings contradict the classical concept of satDNAs organization (as outlined in Introduction). The 11 most abundant satDNAs of *C. gigas* associate with Helitron TEs, indicating their role in satDNA dispersal (Tunjić-Cvitanić et al. 2021). In this work, we observed high level of signal interspersion for the satDNAs related to Helitrons, with localized organization being more of an exception (Figs. 5, 6). This indicates that influence of TE on satDNA distribution and organization extends also to other oyster species. Helitron-related satDNAs contribute substantially to the satellitomes (Fig. 3C), which is consistent with the high abundance of these elements in oyster genomes. Helitrons account for 9.88% of *O. edulis,* 8.74% of *C. virginica,* 12.47% of *C. gigas* (Boutet et al. 2022) and 12.60% of *C. hongkongensis* genome (Li et al. 2020).

Several models aim to explain generation of tandem repeats from TEs (Grabundzija et al. 2016; Hikosaka and Kawahara 2004; McGurk and Barbash 2018; Xiong et al. 2016,). TEs not only serve as origin for satDNA but were proposed to be facilitators/drivers of their dispersal (Cohen et al. 2010; Grabundzija et al. 2016; Hofstatter et al. 2022; Kuhn et al. 2021; Paço et al. 2019; Tunjić-Cvitanić et al. 2021; Zattera and Bruschi 2022). Whether tandem repeats are derived from TEs by tandemization of their parts or TEs capture parts of satDNA arrays and continue propagating them is probably situation- and genome-dependent. Both scenarios could occur simultaneously. Scalvenzi and Pollet (2014) proposed that TEs assimilate repeats and disperse them, and repeats derived from TEs can serve as a basis for generating new satDNAs. TEs overburdened with numerous tandem repeats undergo a decrease in their transposition rate and degeneration of TE components, starting to resemble classical satDNA arrays. In that respect, TE-incorporated, intermediate and standalone arrays could be expected in the genome, as observed for Cg170/HindIII/OYS1 in six oyster species (Table 4; Fig. 5).

This study contributes to our understanding of the extensive connection between satDNAs and TEs documented in bivalve species (Gaffney et al. 2003; Kourtidis et al. 2006; Petraccioli et al. 2015; Plohl et al. 2010; Šatović and Plohl 2013, 2018; Šatović et al. 2016, 2018; Tunjić-Cvitanić et al. 2021). A substantial number of satDNAs constituting the satellitomes of the six oyster species exhibit sequence similarity to different TEs or their components (Supplementary Tables S1–S5, Tunjić-Cvitanić et al. 2021). For numerous satDNAs similarity region encompasses the whole monomer sequence with similarity exceeding 90%. When looking into those where similarity encompasses only segment of the monomer or monomer segments exhibit similarity to different TEs, additional information has to be considered. Multiple insertions of different TEs happen in close proximity, and such loci can serve as hotspots for further insertions (Palomeque et al. 2006). This can lead to the formation of DNA segments that contain short stretches of similarity to different TEs. Furthermore, heterochromatin regions often contain “graveyards” of dead TEs, housing truncated, mutated, rearranged and deteriorated elements. In the oyster *C. gigas* we have revealed the existence of complex loci generated by insertion, deletion, tandemization, and recombination, involving satDNA arrays and Helitron components (Šatović-Vukšić and Plohl 2021). Tandemization of DNA segments in such genomic locations can occur, facilitated by the presence of direct, inverted, or palindromic motifs commonly found therein. Further propagation would result in satDNAs that contain limited stretches of similarity to different TEs (marked as “Multiple TE” in Supplementary Tables S1–S5). We have observed that as the Repbase database undergoes constant updates, satDNAs previously unattributed or showing fragmentary similarities to several repetitive sequences are now being assigned with high similarity and complete length to a specific TE. This would indicate that even more satDNAs are TE-related, but the corresponding TEs have not been characterized yet.

The majority of satDNAs of oysters resemble TEs, both in terms of sequence similarity and chromosomal distribution, questioning whether TE-unrelated satDNAs exist in these genomes. The TE-related, TE-derived or TE-propagated satellitomes, indicate that satDNA sequences are influenced by a larger number of complex mechanisms beyond the gold standards of satDNA evolution (amplification, homogenization, mutation accumulation, and degeneration) (Camacho et al 2022; Garrido-Ramos 2017; Plohl et al. 2008, 2012). For example, the continuous replenishment of genomes with new and identical satDNA monomers from TE-propagated satDNA families may result in the appearance of a great number of highly similar monomer copies, creating an illusion of family conservation (Belyayev et al. 2020). This observation invokes the important question of applicability of the satDNA library model to such experimental systems. The perseverance of OYS1—13 in the genomes of the oyster species (Table 3) would speak in favor of the library. However, Cg170/HindIII (OYS1) displays fluctuation between the standalone and TE-associated form (Table 4; Fig. 5). Furthermore, in situations where entire TEs or substantial parts thereof are tandemized (Fig. 2), such sequences would be more accurately described using the term “TE library”. Therefore, it may be the most appropriate to use the term “repetitive DNA library”, encompassing both the “satDNA library” and “TE library”, when examining repetitive sequences with potential variation in the organizational form and repeat type affiliation across related taxa.

## Conclusions

Oysters possess multiple characteristics that qualify them as valuable non-standard model species for exploring repetitive DNA sequences. This is further enhanced here with information on the novel constitution of the satellitome, the scarcity of TE-unrelated satDNAs, and substantial complexity in studying the satDNA library in these genomes. To understand the repetitive DNA landscape in genomes with such satellitome organization the following must be employed: a series of individual clusterings; comparative clustering; use of a consensus dataset containing satellitome data for all species; and intensive manual curation. Our analysis also highlights the need to expand the terminology on principles that explain evolution of tandem repeats in this particular group of species, as well as in a broader context. We suggest that when a certain repetitive sequence with the potential fluctuation in the organizational form and repeat-type affiliation is studied, the usage of the term “repetitive DNA library”, encompassing both the “satDNA library” and “TE library”, is more appropriate.

### Supplementary Information

Below is the link to the electronic supplementary material.

Supplementary file1 (RAR 18254 KB)

## Data Availability

The data that support the findings of this study are included in this article (and its supplementary information) and are uploaded to the NCBI database with accession numbers provided in this article.

## References

[CR1] Athanasopoulou K, Boti MA, Adamopoulos PG, Skourou PC, Scorilas A (2022). Third-generation sequencing: the spearhead towards the radical transformation of modern genomics. Life.

[CR2] Bao W, Kojima KK, Kohany O (2015). Repbase update, a database of repetitive elements in eukaryotic genomes. Mob DNA.

[CR3] Belyayev A, Josefiová J, Jandová M, Mahelka V, Krak K, Mandák B (2020). Transposons and satellite DNA: on the origin of the major satellite DNA family in the *Chenopodium* genome. Mob DNA.

[CR4] Biscotti MA, Barucca M, Capriglione T, Odierna G, Olmo E, Canapa A (2008). Molecular and cytogenetic characterization of repetitive DNA in the Antarctic polyplacophoran *Nuttallochiton mirandus*. Chromosom Res.

[CR5] Biscotti MA, Olmo E, Heslop-Harrison JS (2015). Repetitive DNA in eukaryotic genomes. Chromosom Res.

[CR6] Boštjančić LL, Bonassin L, Anušić L, Lovrenčić L, Besendorfer V, Maguire I, Grandjean F, Austin CM, Greve C, Hamadou AB, Mlinarec J (2021). The *Pontastacus leptodactylus* (Astacidae) repeatome provides insight into genome evolution and reveals remarkable diversity of satellite DNA. Front Genet.

[CR7] Bouilly K, Chaves R, Leitao A, Benabdelmouna A, Guedes-Pinto H (2008). Chromosomal organization of simple sequence repeats in chromosome patterns. J Genet.

[CR8] Boutet I, Alves Monteiro HJ, Baudry L, Takeuchi T, Bonnivard E, Billoud B, Farhat S, Gonzales-Araya R, Salaun B, Andersen AC, Toullec JY, Lallier FH, Flot JF, Guiglielmoni N, Guo X, Li C, Allam B, Pales-Espinosa E, Hemmer-Hansen J, Moreau P (2022). Chromosomal assembly of the flat oyster (*Ostrea edulis* L.) genome as a new genetic resource for aquaculture. Evol Appl.

[CR9] Cabral-de-Mello DC, Zrzavá M, Kubíčková S, Rendón P, Marec F (2021). The role of satellite DNAs in genome architecture and sex chromosome evolution in Crambidae moths. Front Genet.

[CR10] Cabral-de-Mello DC, Mora P, Rico-Porras JM, Ferretti ABSM, Palomeque T, Lorite P (2023). The spread of satellite DNAs in euchromatin and insights into the multiple sex chromosome evolution in Hemiptera revealed by repeatome analysis of the bug *Oxycarenus hyalinipennis*. Insect Mol Biol.

[CR11] Camacho JPM, Cabrero J, López-León MD, Martín-Peciña M, Perfectti F, Garrido-Ramos MA, Ruiz-Ruano FJ (2022). Satellitome comparison of two oedipodine grasshoppers highlights the contingent nature of satellite DNA evolution. BMC Biol.

[CR12] Charlesworth B, Sniegowski P, Stephan W (1994). The evolutionary dynamics of repetitive DNA in eukaryotes. Nature.

[CR13] Clabby C, Goswami U, Flavin F, Wilkins NP, Houghton JA, Powell R (1996). Cloning, characterization and chromosomal location of a satellite DNA from the Pacific oyster, *Crassostrea gigas*. Gene.

[CR14] Cohen S, Agmon N, Sobol O, Segal D (2010). Extrachromosomal circles of satellite repeats and 5S ribosomal DNA in human cells. Mob DNA.

[CR15] Dias GB, Svartman M, Delprat A, Ruiz A, Kuhn GCSS (2014). Tetris is a foldback transposon that provided the building blocks for an emerging satellite DNA of *Drosophila virilis*. Genome Biol Evol.

[CR16] Dover GA (1986). Molecular drive in multigene families: how biological novelties arise, spread and are assimilated. Trends Genet.

[CR17] Fry K, Salser W (1977). Nucleotide sequences of HS-α satellite DNA from kangaroo rat *Dipodomys ordii* and characterization of similar sequences in other rodents. Cell.

[CR18] Gaffney PM, Pierce JC, Mackinley AG, Titchen DA, Glenn WK (2003). Pearl, a novel family of putative transposable elements in bivalve mollusks. J Mol Evol.

[CR19] Garrido-Ramos MA (2017). Satellite DNA: an evolving topic. Genes.

[CR20] Gomes-dos-Santos A, Lopes-Lima M, Castro LFC, Froufe E (2020). Molluscan genomics: the road so far and the way forward. Hydrobiologia.

[CR21] Grabundzija I, Messing SA, Thomas J, Cosby RL, Bilic I, Miskey C, Gogol-Döring A, Kapitonov V, Diem T, Dalda A, Jurka J, Pritham EJ, Dyda F, Izsvák Z, Ivics Z (2016). A Helitron transposon reconstructed from bats reveals a novel mechanism of genome shuffling in eukaryotes. Nat Commun.

[CR22] Hartley G, O’Neill R (2019). Centromere repeats: hidden gems of the genome. Genes.

[CR23] Hikosaka A, Kawahara A (2004). Lineage-specific tandem repeats riding on a transposable element of MITE in *Xenopus* evolution: a new mechanism for creating simple sequence repeats. J Mol Evol.

[CR24] Hofstatter PG, Thangavel G, Lux T, Neumann P, Vondrak T, Novak P, Zhang M, Costa L, Castellani M, Scott A, Toegelová H, Fuchs J, Mata-Sucre Y, Dias Y, Vanzela ALL, Huettel B, Almeida CCS, Šimková H, Souza G, Pedrosa-Harand A (2022). Repeat-based holocentromeres influence genome architecture and karyotype evolution. Cell.

[CR25] Jurka J, Kapitonov VV, Kohany O, Jurka MV (2007). Repetitive sequences in complex genomes: structure and evolution. Annu Rev Genom Hum Genet.

[CR26] Kojima KK (2019). Structural and sequence diversity of eukaryotic transposable elements. Genes Genet Syst.

[CR27] Kourtidis A, Drosopoulou E, Pantzartzi CN, Chintiroglou CC, Scouras ZG (2006). Three new satellite sequences and a mobile element found inside HSP70 introns of the Mediterranean mussel (*Mytilus galloprovincialis*). Genome.

[CR28] Kuhn GCS, Heringer P, Dias GB, Ugarković Đ (2021). Structure, organization, and evolution of satellite DNAs: insights from the *Drosophila repleta* and *D. virilis* species groups. Satellite DNAs in physiology and evolution.

[CR01] Kumar S, Suleski M, Craig JM, Kasprowicz AE, Sanderford M, Li M, Stecher G, Hedges SB (2022). TimeTree 5: an expanded resource for species divergence times. Mol Biol Evol.

[CR29] Langdon T, Seago C, Mende M, Leggett M, Thomas H, Forster JW, Jones RN, Jenkins G (2000). Retrotransposon evolution in diverse plant genomes. Genetics.

[CR30] Li Y, Nong W, Baril T, Yip HY, Swale T, Hayward A, Ferrier DEK, Hui JHL (2020). Reconstruction of ancient homeobox gene linkages inferred from a new high-quality assembly of the Hong Kong oyster (*Magallana hongkongensis*) genome. BMC Genom.

[CR31] Li C, Kou Q, Zhang Z, Hu L, Huang W, Cui Z, Liu Y, Ma P, Wang H (2021). Reconstruction of the evolutionary biogeography reveal the origins and diversification of oysters (Bivalvia: Ostreidae). Mol Phylogenet Evol.

[CR32] López-Flores I, Garrido-Ramos MA, Garrido-Ramos MA (2012). The repetitive DNA content of eukaryotic genomes. Genome dynamics.

[CR33] López-Flores I, de la Herrán R, Garrido-Ramos MA, Boudry P, Ruiz-Rejón C, Ruiz-Rejón M (2004). The molecular phylogeny of oysters based on a satellite DNA related to transposons. Gene.

[CR34] Lower SS, McGurk MP, Clark AG, Barbash DA (2018). Satellite DNA evolution: old ideas, new approaches. Curr Opin Genet Dev.

[CR35] Luchetti A (2015). TerMITEs: miniature inverted-repeat transposable elements (MITEs) in the termite genome (Blattodea: Termitoidae). Mol Genet Genom.

[CR36] Macas J, Koblízková A, Navrátilová A, Neumann P (2009). Hypervariable 3’ UTR region of plant LTR-retrotransposons as a source of novel satellite repeats. Gene.

[CR02] Martínez-Expósito MJ, Pasantes JJ, Méndez J (1994). NOR activity in larval and juvenile mussels (*Mytilus galloprovincialis* Lmk.). J Exp Mar Bio Ecol.

[CR37] Mascagni F, Barghini E, Ceccarelli M, Baldoni L, Trapero C, Díez CM, Natali L, Cavallini A, Giordani T (2022). The singular evolution of *Olea* genome structure. Front Plant Sci.

[CR38] McGurk MP, Barbash DA (2018). Double insertion of transposable elements provides a substrate for the evolution of satellite DNA. Genome Res.

[CR39] Negm S, Greenberg A, Larracuente AM, Sproul JS (2021). RepeatProfiler: a pipeline for visualization and comparative analysis of repetitive DNA profiles. Mol Ecol Resour.

[CR40] Novák P, Neumann P (2020). Global analysis of repetitive DNA from unassembled sequence reads using RepeatExplorer2. Nat Protoc.

[CR41] Novák P, Neumann P, Macas J (2010). Graph-based clustering and characterization of repetitive sequences in next-generation sequencing data. BMC Bioinform.

[CR42] Novák P, Neumann P, Pech J, Steinhaisl J, Macas J (2013). RepeatExplorer: a galaxy-based web server for genome-wide characterization of eukaryotic repetitive elements from next-generation sequence reads. Bioinformatics.

[CR43] Novák P, Robledillo LÁ, Koblížková A, Vrbová I, Neumann P, Macas J (2017). TAREAN: a computational tool for identification and characterization of satellite DNA from unassembled short reads. Nucl Acids Res.

[CR44] Paço A, Freitas R, Vieira-Da-Silva A (2019). Conversion of DNA sequences: from a transposable element to a tandem repeat or to a gene. Genes.

[CR45] Palomeque T, Carrillo JA, Muñoz-López M, Lorite P (2006). Detection of a mariner-like element and a miniature inverted-repeat transposable element (MITE) associated with the heterochromatin from ants of the genus *Messor* and their possible involvement for satellite DNA evolution. Gene.

[CR46] Peñaloza C, Gutierrez AP, Eory L, Wang S, Guo X, Archibald AL, Bean TP, Houston RD (2021). A chromosome-level genome assembly for the Pacific oyster (*Crassostrea gigas*). Gigascience.

[CR47] Pérez-García C, Morán P, Pasantes JJ (2011). Cytogenetic characterization of the invasive mussel species *Xenostrobus securis* Lmk. (Bivalvia: Mytilidae). Genome.

[CR48] Petraccioli A, Odierna G, Capriglione T, Barucca M, Forconi M, Olmo E, Biscotti MA (2015). A novel satellite DNA isolated in *Pecten jacobaeus* shows high sequence similarity among molluscs. Mol Genet Genomics.

[CR49] Pita S, Panzera F, Mora P, Vela J, Cuadrado Á, Sánchez A, Palomeque T, Lorite P (2017). Comparative repeatome analysis on *Triatoma infestans* Andean and Non-Andean lineages, main vector of Chagas disease. PLoS ONE.

[CR50] Plohl M, Luchetti A, Mestrović N, Mantovani B (2008). Satellite DNAs between selfishness and functionality: structure, genomics and evolution of tandem repeats in centromeric (hetero)chromatin. Gene.

[CR51] Plohl M, Petrović V, Luchetti A, Ricci A, Šatović E, Passamonti M, Mantovani B (2010). Long-term conservation vs high sequence divergence: the case of an extraordinarily old satellite DNA in bivalve mollusks. Heredity.

[CR52] Plohl M, Meštrović N, Mravinac B, Garrido-Ramos MA (2012). Satellite DNA evolution. Genome dynamics.

[CR53] Robledo JAF, Yadavalli R, Allam B, Pales-Espinosa E, Gerdol M, Greco S, Stevick RJ, Gómez-Chiarri M, Zhang Y, Heil CA, Tracy AN, Bishop-Bailey D, Metzger MJ (2018). From the raw bar to the bench: bivalves as models for human health. Dev Comp Immunol.

[CR54] Ruiz-Ruano FJ, López-León MD, Cabrero J, Camacho JPM (2016). High-throughput analysis of the satellitome illuminates satellite DNA evolution. Sci Rep.

[CR55] Šatović E, Plohl M (2013). Tandem repeat-containing MITE elements in the clam *Donax trunculus*. Genome Biol Evol.

[CR56] Šatović E, Plohl M (2018). Distribution of DTHS3 satellite DNA across 12 bivalve species. J Genet.

[CR57] Šatović E, Vojvoda Zeljko T, Luchetti A, Mantovani B, Plohl M (2016). Adjacent sequences disclose potential for intra-genomic dispersal of satellite DNA repeats and suggest a complex network with transposable elements. BMC Genom.

[CR58] Šatović E, Vojvoda Zeljko T, Plohl M (2018). Characteristics and evolution of satellite DNA sequences in bivalve mollusks. Eur Zool J.

[CR59] Šatović E, Tunjić Cvitanić M, Plohl M (2020). Tools and databases for solving problems in detection and identification of repetitive DNA sequences. Period Biol.

[CR60] Šatović Vukšić E, Plohl M, Ugarković Đ (2021). Exploring satellite DNAs: specificities of bivalve mollusks genomes. Satellite DNAs in physiology and evolution.

[CR61] Šatović-Vukšić E, Plohl M (2021). Classification problems of repetitive DNA sequences. DNA.

[CR62] Šatović-Vukšić E, Plohl M (2023). Satellite DNAs—from localized to highly dispersed genome components. Genes.

[CR63] Scalvenzi T, Pollet N (2014). Insights on genome size evolution from a miniature inverted repeat transposon driving a satellite DNA. Mol Phylogenet Evol.

[CR64] Schmidt T, Heslop-Harrison JS (1998). Genomes, genes and junk: the large-scale organization of plant chromosomes. Trends Plant Sci.

[CR65] Sedlazeck FJ, Lee H, Darby CA, Schatz MC (2018). Piercing the dark matter: bioinformatics of long-range sequencing and mapping. Nat Rev Genet.

[CR66] Sharma A, Wolfgruber TK, Presting GG (2013). Tandem repeats derived from centromeric retrotransposons. BMC Genom.

[CR67] Suárez-Ulloa V, Fernández-Tajes J, Manfrin C, Gerdol M, Venier P, Eirín-López JM (2013). Bivalve omics: state of the art and potential applications for the biomonitoring of harmful marine compounds. Mar Drugs.

[CR68] Tek AL, Song J, Macas J, Jiang J (2005). Sobo, a recently amplified satellite repeat of potato, and its implications for the origin of tandemly repeated sequences. Genetics.

[CR69] Thakur J, Packiaraj J, Henikoff S (2021). Sequence, chromatin and evolution of satellite DNA. Int J Mol Sci.

[CR70] Thomas J, Pritham EJ (2015). *Helitrons*, the eukaryotic rolling-circle transposable elements. Microbiol Spectr.

[CR71] Tunjić Cvitanić M, Vojvoda Zeljko T, Pasantes JJ, García-Souto D, Gržan T, Despot-Slade E, Plohl M, Šatović E (2020). Sequence composition underlying centromeric and heterochromatic genome compartments of the Pacific oyster *Crassostrea gigas*. Genes.

[CR72] Tunjić-Cvitanić M, Pasantes JJ, García-Souto D, Cvitanić T, Plohl M, Šatović-Vukšić E (2021). Satellitome analysis of the Pacific oyster *Crassostrea gigas* reveals new pattern of satellite DNA organization, highly scattered across the genome. Int J Mol Sci.

[CR73] Vojvoda Zeljko T, Pavlek M, Meštrović N, Plohl M (2020). Satellite DNA - like repeats are dispersed throughout the genome of the Pacific oyster *Crassostrea gigas* carried by *Helentron* non-autonomous mobile elements. Sci Rep.

[CR74] Vondrak T, Robledillo ÁL, Novák P, Koblížková A, Neumann P, Macas J (2020). Characterization of repeat arrays in ultra-long nanopore reads reveals frequent origin of satellite DNA from retrotransposon-derived tandem repeats. Plant J.

[CR75] Xiong W, Dooner HK, Du C (2016). Rolling-circle amplification of centromeric *Helitrons* in plant genomes. Plant J.

[CR76] Zattera ML, Bruschi DP (2022). Transposable elements as a source of novel repetitive DNA in the eukaryote genome. Cells.

[CR77] Zhang G, Fang X, Guo X, Li L, Luo R, Xu F, Yang P, Zhang L, Wang X, Qi H, Xiong Z, Que H, Xie Y, Holland PWH, Paps J, Zhu Y, Wu F, Chen Y, Wang J, Peng C (2012). The oyster genome reveals stress adaptation and complexity of shell formation. Nature.

